# Biocompatibility of crystalline opal nanoparticles

**DOI:** 10.1186/1475-925X-11-78

**Published:** 2012-10-22

**Authors:** Marlen Hernández-Ortiz, Laura S Acosta-Torres, Genoveva Hernández-Padrón, Alicia I Mendieta, Rodolfo Bernal, Catalina Cruz-Vázquez, Victor M Castaño

**Affiliations:** 1 Programa de Posgrado en Ciencia de Materiales del, Universidad de Sonora, A P 130, Hermosillo, Sonora, 83000, México; 2 Departamento de Investigación en Polímeros y Materiales, Universidad de Sonora, A P 130, Hermosillo, Sonora, 83000, México; 3 Escuela Nacional de Estudios Superiores, Universidad Nacional Autónoma de México, Unidad León, Boulevard UNAM No. 2011 Predio el Potrero y el Saucillo, C.P. 36969, León, Guanajuato, México; 4 Departamento de Nanotecnología, Campus Juriquilla, Querétaro, 76230, México; 5 Departamento de Ingeniería Molecular de Materiales, Centro de Física Aplicada y Tecnología Avanzada, Campus Juriquilla, Querétaro, 76230, México; 6 Instituto de Neurobiología, Universidad Nacional Autónoma de México, Campus Juriquilla, Querétaro, 76230, México; 7 Departamento de Investigación en Física, Universidad de Sonora, A. P. 5-088, Hermosillo, Sonora, 83190, México; 8 Centro de Física Aplicada y Tecnología Avanzada, Universidad Nacional Autónoma de México, Campus Juriquilla, Querétaro, 76230, México

**Keywords:** Cytotoxicity, Genototoxicity, Synthetic opal, BrdU and MTT assay

## Abstract

**Background:**

Silica nanoparticles are being developed as a host of biomedical and biotechnological applications. For this reason, there are more studies about biocompatibility of silica with amorphous and crystalline structure. Except hydrated silica (opal), despite is presents directly and indirectly in humans. Two sizes of crystalline opal nanoparticles were investigated in this work under criteria of toxicology.

**Methods:**

In particular, cytotoxic and genotoxic effects caused by opal nanoparticles (80 and 120 nm) were evaluated in cultured mouse cells via a set of bioassays, methylthiazolyldiphenyl-tetrazolium-bromide (MTT) and 5-bromo-2′-deoxyuridine (BrdU).

**Results:**

3T3-NIH cells were incubated for 24 and 72 h in contact with nanocrystalline opal particles, not presented significant statistically difference in the results of cytotoxicity. Genotoxicity tests of crystalline opal nanoparticles were performed by the BrdU assay on the same cultured cells for 24 h incubation. The reduction of BrdU-incorporated cells indicates that nanocrystalline opal exposure did not caused unrepairable damage DNA.

**Conclusions:**

There is no relationship between that particles size and MTT reduction, as well as BrdU incorporation, such that the opal particles did not induce cytotoxic effect and genotoxicity in cultured mouse cells.

## Background

Technology is now available to produce nanoparticles materials, with a wide range of size distributions, shapes and modified surface functions. Particularly, silicon nanoparticles, are of interest as a biomarker because they are potentially bioinert and do not require a thick protective shell [1-4]. In addition, silica nanoparticles are being developed as a host of biomedical and biotechnological applications such as cancer therapy, DNA transfection, drug delivery and enzyme immobilization [5-7].

The extensive use of nanomaterials has promoted the study of nanotoxicology in parallel to the development of applications. This is due to the fact that the surfaces of biomaterials (e.g., implants and medical devices) are immediately covered by biomolecules (e.g., proteins, natural organic materials, detergents, and enzymes) as they come into contact with a biological medium. Nanoparticles coated with proteins have a conformation that may be disrupted or induced to aggregate, which, in turn, can trigger unexpected cellular responses. The key role of protein-nanoparticle interactions in nanomedicine and nanotoxicity has begun to emerge recently via the identification of the nanoparticle and protein corona. This dynamic layer of proteins and/or other biomolecules adsorbed to the nanoparticle surface determines how a nanoparticle interacts with living systems and thereby modifies the cellular responses [8-11].

The affinity and amount of proteins adsorbed on the surfaces of nanoparticle are highly dependent on the nanomaterial composition, size and surface chemistry [10]. For example, the nano-sized silica can generate adverse effects, like liver injury and inflammation; and the exposure of amorphous spherical silicon dioxide nanoparticles of different sizes induces decreases in viability of human endothelial cells, an expression of its cytotoxicity which was apparently dependent of the particle size. Besides, each organ has different reactions; some studies indicate that nano-silica can cause cytotoxicity and primary damage in DNA but not mutagenicity in cultured mammalian cells [2,6,12-16].

Specifically, a polymorphous of the silica, called opal, has not been studied regarding its biocompatibility. Opal is a natural hydrous silica with either amorphous (opal-A) or ordered cristobalite structures (opal-C) and a spherical shape over a wide range of diameters from several tens of nanometers to various micrometers [17-19]. Humans are exposed directly and indirectly to opals. For example, some animals and plants contain opal as a cross-linking agent in connective tissues [20,21] and the infiltration of biomolecules in photonic crystals to get improved luminescence spectra of DNA infiltrated opal due to the possible formation of new photon-electron bound states [22]. The last study was made *in vitro* and does not consider toxicity tests. For this reason, the aim of the present work is to investigate *in vitro* whether nanocrystalline opal synthetized by sedimentation method could induce cytotoxic and genotoxic effects in 3T3-NIH mouse epithelial cells, using MTT and BrdU assays, respectively.

### Cytotoxicity of magnetic nanoparticles

Advancements in nanotechnology over the last 20 years have led to the development of novel magnetic nanoparticles. The general theory of nuclear relaxation in the presence of paramagnetic substances is based on a monocrystalline or polycrystalline iron oxide core with a diameter of 5–30 nm embedded within a polymer coating [23]. Superparamagnetic iron oxide nanoparticles (SPIONs) are used in a large variety of biomedical applications, such as cell labeling, hyperthermia, controlled drug delivery, *in vivo* cell tracking, hyperthermia, treatment of cancer, as magnetic resonance imaging (MRI) contrast agents, including [24-27]. Among all types of nanoparticles, SPIONs are the most promising candidates for use as contrast agents not only for their suitable magnetic saturation and superparamagnetic properties, but also due to their colloidal stability and biocompatibility [23,24,26,28,29].

In summary, cytotoxity of magnetic nanoparticles is strongly dependent of their chemistries and physics characteristic, cell lines, cell cycle and examined technique. The manner in which SPIONs are chemically modified will indeed impact cytotoxicity outcomes *in vitro* and also the toxicokinetics and dynamics *in vivo*[23]. Cytotoxicity of the bare and coated SPIONs has been assessed via various methods such as the cell-life cycle assay, MTT assay, comet assay, TUNEL assay (i.e., for apoptosis detection), and several *in vivo* models [28].

The relation with this paper is based in that mesoporous silica is the magnetic carrier in whose pores is adsorbed the drug [24]. Specifically, silica has also received tremendous attention given its long industrial history as an occupational carcinogen. Also nanoscale silica can disrupt nuclear integrity by forming intranuclear protein aggregates that can lead to inhibition of replication, transcription and cell proliferation, as reported by Chen *et al.*[26].

## Methods

### Production and characterization of opal nanoparticles

Synthetic opals were produced by Stöber technique [30]. Nanodispersive silica spheres near 80 and 120 nm in diameter were synthesized through the sol-gel process of tetraethyl orthorsilicate (TEOS; Si (OC_2_H_5_)_4_) in a water-ethanol solution with presence of ammonium hydroxide as a catalyst, each particle size corresponds at a batch called O1 and O2, respectively. The colloid solutions were dried at 60°C. Finally, samples were sintered to 1150°C for 2 days with extreme sluggishness.

The structure and the morphology of samples were characterized by X-Ray Diffraction (XRD), using a Rigaku diffractometer, model Miniflex, with Cu Kα radiation, and Scanning Electron Microscopy (SEM) analysis, obtained with a JEOL JSM-6060LV microscope using gold as coater.

The purity of opal nanoparticles was proved with infrared spectroscopy (FTIR), using a Perkin-Elmer Spectrum GX by the KBr pellet method; and a chemical analysis by energy-dispersive spectroscopy (EDS) was developed in a JEOL JSM-5410LV Scanning Microscope.

### Preparation of opal nanoparticles

Opals were added into 15 mL acid mixture of H_2_SO_5_:HNO_3_ (3:1) in an ultrasonic bath for 24 h, then in 0.1 M NaOH aqueous solution at 90°C for 2 h, finally in 0.1 M HCl aqueous solution at 90°C for 2 h. Thereafter, the treated opals were washed with distilled water and then centrifuged and dried. After drying, the particles were dissolved in distilled water and the solution was sonicated for 20 min before the cell evaluations.

### Cell line and culture

The 3T3-NIH mouse fibroblast were cultured in DMEM (Dulbecco’s Modified-Eagle medium) supplemented with FBS (Fetal Bovine Serum, 10%) and penicillin-streptomycin (1%). The mouse fibroblasts were seeded in 24-well plates (1 x 10^4^ cells) during 24 h, and then the medium was renewed and the experimental opals samples were put in contact with cells.

### Cytotoxicity assay

The cytotoxicity of particles was determined using the MTT assay [31]. Briefly, opal particles were set in contact with cells and incubated in a volume of 500 μL into each well of a 24-well plate during 24 and 72 h. After each period of time, the MTT reagent was added to each well at a final concentration of 0.5 mg/mL and plates were incubated at 37°C for 40 min. Next, a solubilizing solution was added to each well and mixed thoroughly during 5 min. The optical density (OD) was read on a spectrophotometric ELISA plate reader at a wavelength of 570 nm. Cells not in contact with opals nanoparticles were used as control group. The results of the experiments were expressed as percentage of cell viability.

### Genotoxicity assay

The quantitative determination of DNA synthesis in cells was measured using a modified BrdU test. After the cell incubation for 24 h, the BrdU labeling reagent was added and the cells were incubated again for further 4 h. During this period, the thymidine analogue BrdU is incorporated instead of thymidine into the DNA of proliferating cells. After removing the culture medium, the cells were fixed and the DNA was denatured in one step by adding FixDenat. The quantity of BrdU incorporation was detected by the monoclonal antibody from mouse hybrid cells conjugated with peroxidase (anti-BrdU-POD) which binds to the BrdU in newly synthesized cellular DNA. These immune complexes were detected by the subsequent substrate reaction and after addition of the stop solution (1 mol/L H_2_SO_4_) the colorimetric measurement of BrdU was started with the respective wavelength 450 nm in an ELISA microreader. The color intensity and thereby the absorbance directly correlated to the amount of DNA synthesis and hereby to the number of proliferating cells. The percentage of BrdU incorporation was determined by the analysis of cells treated with test substances, compared to controls.

### Statistical analysis

The MTT and BrdU experiments were run using n = 3 on each group. Statistical analysis was carried out using Student’s *t*-test and Mann Whitney test respectively. Differences were considered significant when the P value was less than 0.05.

## Results and discussion

### Characterization of the opal nanoparticles

**Figure 1:**
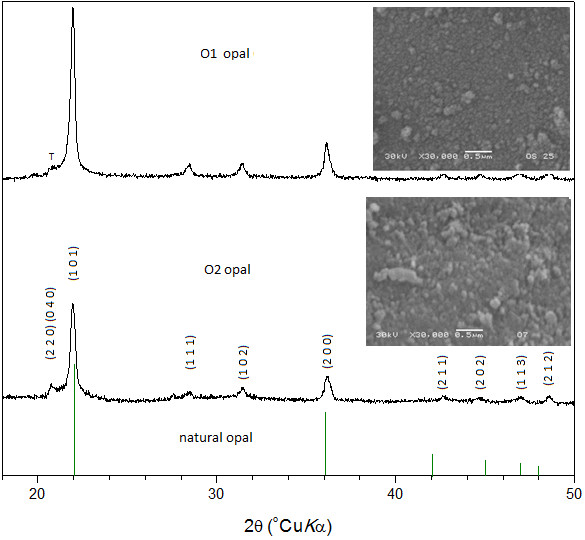
**XRD pattern and SEM micrographs of O1 and O2 opals. **The lines present the band features of natural opal as reference and peaks marked T to tridymitic stacking.

Figure 1 presents SEM images and XRD patterns of the O1 and O2 opals. The images illustrate spherical particles in the opals with heterogeneous size distribution that indicate an average size around 80 and 120 nm, respectively. Size established for “secondary” particles which are considered as spherical particles smaller of the relatively large particles called agglomerates or “tertiary” structures. The secondary particles consist of smaller primary spherical particles 5–10 nm in diameter, which not seen in this case by the limitation of the technique [32]. The XRD patterns of all specimens show in particular a outstanding peak at ~19–24° 2θ with full-width half-maximum (FWHM) <5.6° and a peak at ~36° 2θ dominant features of the opal-C [33-35]. 

**Figure 2:**
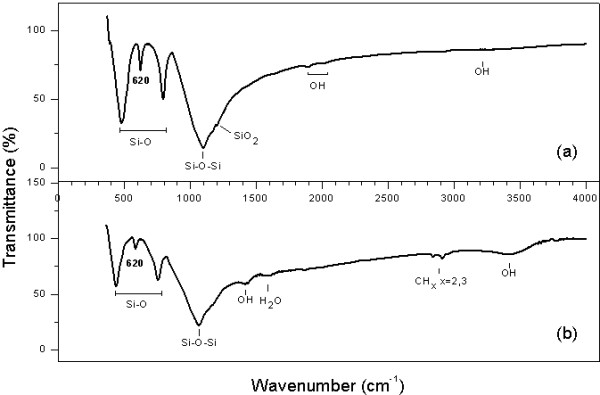
**FTIR transmittance spectra of opals nanoparticles: a) O1 and b) O2. **Identification of characteristic absorption band at 620 cm^−1^, attributed to the crystalline opal (opal-C) for both samples.

The FTIR spectra of all samples are illustrated in Figure 2. The typical IR spectrum of opal-C indicates an absorbance band at 620 cm^−1^ which is characteristic for low-cristobalite is shown in O1 opal (Figure 2a) and O2 opal (Figure 2b). Furthermore, the remaining absorption bands near 1100, 790, and 480 cm^−1^ are common to all silicates with tetrahedrally coordinated silicon. Both samples present broad band assigned to the O–H stretching (3500 cm^−1^, 1450 cm^−1^ and between 1800 and 2000 cm^−1^) and band due to scissor bending vibration of molecular water (1630 cm^−1^), better defined for O2 opal. This sample has C–H_2_ and C–H_3_ absorbance group (2930 and 2980 cm^−1^, respectively) corresponding to the presence of unreacted TEOS in the silica particles, its intensity decreases with increasing ageing time. In fact, synthetic opals obtained not present quartz, revealed by the distinctive absorption band absence at 695 cm^−1^[35,36]. 

**Figure 3:**
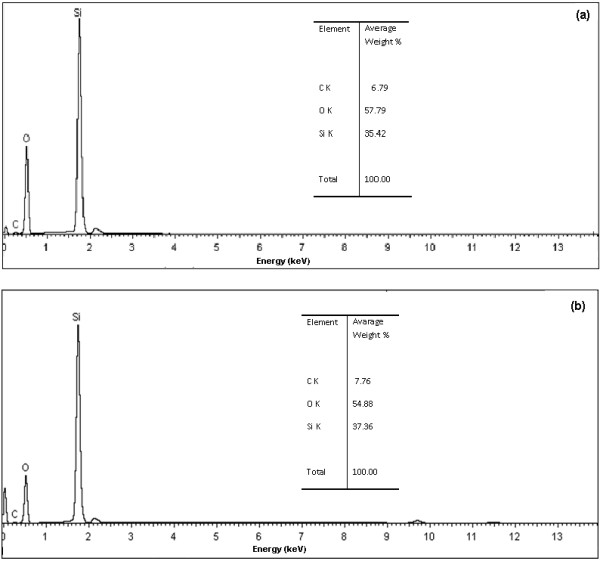
**EDS spectrum from the nanocrystalline particles: a) O1 opal and b) O2 opal. **The chemical analysis of opal nanoparticles shows a composition of silicon and oxygen, with a few concentration of carbon of unreacted TEOS.

In addition to identify the type of opal used, by FTIR; the chemical analysis by EDS presents the purity of both O1 and O2 opal nanoparticles, which is shown in Figure 3 (a) and (b), respectively. The samples exhibit a composition of silicon and oxygen with major proportion in weight (wt%); however, the presence of carbon indicates amounts of 6.79 wt% y 7.76 wt% in O1 and O2 opals, respectively. This confirms that samples contain C–H groups or non-hydrolyzed ethoxy groups (CH_3_CH_2_-O). Several natural opals included in further investigations contain impurities (no-volatile) of less than 0.5 wt% [37]. However, FTIR and XRD show clearly that both samples are opal type C. Moreover, a study prove that the FTIR spectrum of fire opal (natural opal) indicates absorption bands related with C–H groups [38] and other work confirms carbon presence with chemical analysis of different natural opals, but this was attributed to some organic component of the window X-ray detector [39]. 

### Induction of cytotoxicity and of DNA strand breaks by opal

**Figure 4:**
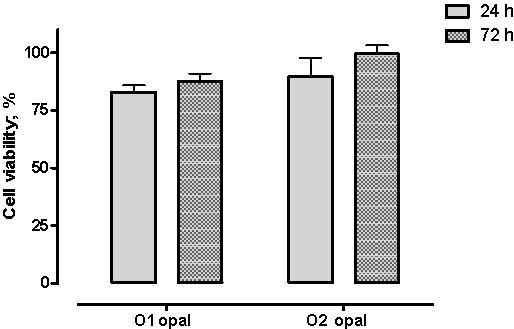
**Effect of opal nanoparticles on MTT reduction. **Significant statistically differences not were found between groups at P > 0.05.

Then, two different size opal particles of nanometric order were tested under identical experimental conditions to clarify the role of particle size in cytotoxicity/cell viability. Figure 4 summarizes the percent cell viability after 24 and 72 h exposed to opal nanoparticles. Spectrophotometric plate experiments where the concentration of opal powders was 0.25 μg/mL presented as a percentage of live cells incubated in O1 opal was about 80 and 85% and the cells viability in O2 opal was about 90 and 98%, to each time respectively. There was an inverse relationship between particle size and MTT reduction; the opal particles less than 100 nm (O1 opal) induced major MTT reductions. Moreover, the test showed that the viability of the cells decreased when the exposure time of synthetic crystalline opal nanoparticles increased. The results indicate there are no significant statistically difference (P > 0.05) between both opals nanoparticles groups during the two evaluated periods of time, indicating less cytotoxic effect toward 3T3-cells. 

**Figure 5:**
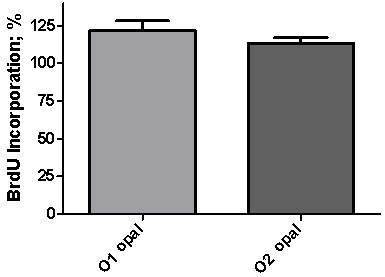
**Histogram of BrdU incorporation in 3T3-NIH cells. **The percentage of BrdU incorporation into the cells indicates that nanocrystalline opal exposure does not show significant difference between the evaluated particles (P = 0.0931).

On the other hand, opal nanoparticles of different sizes were tested to assess the possibility of nanosize-dependent genotoxic effect and the results are showed in Figure 5. The percentage of incorporated BrdU into the exposed 3T3 cells were translated to a minor DNA damage of the cells. The BrdU reactive is an analog of the thymidine nucleotide which is substituted by the thymidine during the replication process (synthesis of DNA), so when the DNA is damaged, the BrdU cannot be incorporated to the DNA chain during replication process indicating a genotoxic effect. In the present study, the percentage of BrdU incorporation into the cells indicates that nanocrystalline opal exposure did not caused unrepairable DNA damage, such as strand breaks and alkali-labile sites, and its behavior is more marked for O1 opal corresponding at smaller size of nanoparticles, showing no significant difference between nanocrystalline particles of opals evaluated (P = 0.0931). 

It observed major grade of cytotoxicity and genotoxicity in the O1 opal with less particle size and low concentration of groups OH and H_2_O molecules (Figure 2). This is important regarding a detailed discussion of the surface area interaction of links OH of the opal with the cellular environment. Some studies support that the smaller silica nanoparticles with greater specific surface area show more toxic effects [5,40]. Indeed, there is an inverse relationship between particle size and number of surface expressed molecules, because the number of atoms or molecules on the surface of the particle may determine the material reactivity [8]. In addition, silanol (SiOH) groups present on the surface of silica particles are capable of forming hydrogen bonds with oxygen and nitrogen groups found in biologic cell membranes, which then may lead to a loss of membrane structure, lysosomal leakage and tissue damage [11]. On the other hand, Costa *et al.* showed that smaller silica particles are not as extensively cross-linked, as observed from the large radius increase within liquid, due to swelling with water and ethanol; also, by its lower concentrations of ammonia, used in the synthesis, contain broadly distributed residual ethoxy groups on surfaces but also largely within the particles [41]. In fact, the oxidative state of nanomaterials at the interface is another potential design feature that can be used to mitigate cytotoxicity [22]. This case could be reason that the O1 opal does not present cell damage significant statistically.

Due to practically there are not studies about toxicity of crystalline opal nanoparticles, results observed of this work are compared with different crystalline and amorphous silica materials on distinct types of cells. This comparison indicates similitude in it exhibited [6,7,42-44]. Dusts composed of amorphous silica (opal), with the exception of fiberglass, are not generally considered to be harmful to humans. On the other hand, a research data suggests that there is fibrogenic activity of different forms of free silica; the action of fused silica, quartz, cristobalite and tridymite on the liver of mice [15]. More even, crystalline silica shows cytotoxicity and genotoxicity based on *in vitro* testing [2], apparently contradicts the work of this paper. Although, the water is considered as active agent, a study shows as the stishovite (crystalline form of silica) diluted in H_2_O could be even more toxic than quartz [21]. Therefore, the water attached to the surface of crystalline opal nanoparticles is an important factor in the interaction process between cells and particles, which influences to avoid toxicity. Also, the concentration of crystalline opal nanoparticles is an important parameter in biocompatibility studies. In the present study, the concentration of sample used is greater than it what might be used in practice; which indicates no damage cytotoxic or genotoxic in cells. Certainly, the physical and chemical effects observed in the interaction between surface and biological environment requires further investigation in order to provide a good physicochemical explanation for the shown phenomenon.

## Conclusions

Within the limitation of the present study we conclude that crystalline opals nanoparticles with 80 to 120 nm in diameter were no cytotoxic and genotoxic to the exposed mouse fibroblast cells.

## Competing interests

The authors declare that they have no competing interests.

## Authors’ contributions

MHO, LSAT and VMC have made substantial contributions to the conception and design of the investigation. LSAT and IM were involved in the data collection. All authors read and approved the final manuscript.
